# Advanced Electrochemical and Opto-Electrochemical Biosensors for Quantitative Analysis of Disease Markers and Viruses

**DOI:** 10.3390/bios12050296

**Published:** 2022-05-04

**Authors:** Najmeh Karimian, Federico Polo, Paolo Ugo

**Affiliations:** 1 Department of Molecular Sciences and Nanosystems, University Ca’ Foscari of Venice, Via Torino 155, 30172 Mestre-Venezia, Italy; federico.polo@unive.it (F.P.); ugo@unive.it (P.U.); 2 European Centre for Living Technology (ECLT), Ca’ Bottacin, Dorsoduro 3911, 30124 Venice, Italy

Instrumental laboratory methods for biochemical and chemical analyses have reached a high level of reliability with excellent sensitivity and specificity. However, the complex sample preparation, the need for trained personnel as well as the use of sophisticated and expensive instrumentation still represent a drawback. Despite the indispensability of laboratory methods that guarantee excellence with regard to their analytical reliability, the need to carry out quantitative analyses for large-scale and rapid screening purposes has prompted the development of novel analytical tools, such as biosensors, capable of providing accurate and precise analytical information at low cost, ease of use and applicability for decentralized monitoring and point-of-care testing [1,2]. The recent global health crisis caused by the COVID-19 pandemic has further alerted the world to the urgent need for rapid and reliable analytical devices, which are suitable for the detection of biologically active components of clinical relevancy.

Especially for the case of immunological tests, this recent experience has highlighted the gap between, e.g., classical ELISA, which provides quantitative and reliable responses, although it must be performed in a centralized laboratory by trained personnel, and strip lateral flow tests with visual detection, suitable for decentralized use, although capable to provide only qualitative information, sometimes with not ideal sensitivity and specificity. New analytical devices are therefore highly sought to fill this gap, which must combine the capability of providing quantitative instrumental responses with fast and user-friendly applicability.

The advantages of electrochemical and optical biosensors (low cost and easy transduction) are nowadays complemented in terms of improved sensitivity by combining electrochemistry (EC) with optical techniques such as electrochemiluminescence (ECL), EC/surface-enhanced Raman spectroscopy (SERS) and EC/surface plasmon resonance (SPR) [3]. Despite recent impressive technological progress in this area, there are still pitfalls that must be overcome to progress towards real world applicability. To this goal, biosensors need to become self-contained and usable automatically or semi-automatically, while guaranteeing precise chemical information and analytical reliability. Hopefully, advances to reach these difficult tasks can derive from the continuous development and application of informatics tools, such as machine learning-based methodologies [4,5]. However, progress in the design of the biosensors’ architecture will also be crucial.

The present Special Issue focuses on some of the abovementioned challenges, while proposing novel approaches and exploring new applications in electrochemical, optical and opto-electrochemical biosensors. In particular, the focus is on the quantitative detection of disease markers and viruses. To this aim, this Special Issue presents both original research articles and authoritative review papers. 

Interesting features of SPR sensors include label-free detection, high sensitivity, real-time monitoring and applicability to raw sample [6,7]. In their contribution to this Special Issue, Masson and coworkers present the new possibility of exploiting SPR to develop a so-called doxy-AuNPs-based SPR biosensor for the fast and sensitive detection of doxycycline [8]. Indeed, the authors demonstrate that the drug doxycycline can tune the formation of Au nanoparticles to enhance the response of a SPR biosensor to this drug. Among other interesting features, it is worth mentioning that the resulting SPR signal scales directly with the concentration of doxycycline, contrary to the usual SPR-based immunosensors which, being based on competition assays, result in a progressive lowering of the response while increasing the analyte concentration. 

In their article, Campuzano, Pingarron and coworkers [9] present a novel amperometric immunosensor for the determination of ST2, a member of the interleukin 1 receptor family. ST2 has well-known relations with inflammatory and other diseases so that high levels of sST2 are found in the serum of patients suffering from several disorders. The proposed method is based on a sandwich immunoassay with electrochemical detection, using a disposable screen-printed carbon electrode. Magnetic immunoconjugates are built on the surface of carboxylic acid-microsized magnetic particles. Captured ST2 is finally sandwiched by a biotinylated secondary antibody conjugated with a streptavidinated peroxidase. The sensor shows interesting selectivity and sensitivity, allowing the determination of soluble ST2 in plasma samples from healthy individuals and patients diagnosed with pancreatic ductal adenocarcinoma (PDAC), with the advantage of a relatively short analysis time (45 min). The good correlation of the obtained results with those provided by classical ELISA demonstrates the potential of the developed strategy for the early diagnosis and/or prognosis of the fatal PDAC disease.

On the stream line of biosensors based on enzyme inhibition effects [10], Worden and coworkers [11] study a novel electrochemical biosensor to detect acetylcholinesterase (AChE) inhibitors that may trigger neurological diseases. The research is based on an integrated approach, combining experimental results within the frame of a theoretical model suitable to be applied to the case of an inhibition-based bi-enzyme (IBE) sensor. Experimentally, AChE and tyrosinase (Tyr) are co-immobilized on the working electrode component of a screen-printed electrode array. Using redox recycling of the electroactive component of the sensor, an amplification mechanism is suitably activated to increase the signal and the sensitivity. The theoretical model is validated by comparison between experimental and calculated results. The model shows its capability to reproduce different trends in the experimental results, ranging from steady-state responses to unsteady-state dynamics of the biosensor, e.g., following the addition of a reactant (phenylacetate) and an ACE inhibitor (PMSF).

In their research article, Kaushik and coworkers [12] present an electrochemical immunosensor for collagen I, chosen as a biomarker for monitoring the regeneration of damaged connective tissue in tendons and ligaments. The proposed immunosensor is fabricated using a self-assembled monolayer (SAM) of a bis-thiol to immobilize gold nanoparticles (AuNPs) which are functionalized with the capture agent, that is a half-reduced monoclonal antibody. Detection is performed by electrochemical impedance spectroscopy and/or SPR, the former technique providing the lower detection limits. Interestingly, the sensor shows improved sensitivity with respect to the commonly used ELISA procedure for the same analysis.

In diagnostics and theranostics, a key issue concerns the capability of detecting cancer biomarkers. Such a role is played by microRNA (miRNA), which are small RNA sequences (18–25 mers), whose expression level is correlated with the onset and development of diseases, including cancer, diabetes and heart disease. In their review article, Amine and coworkers [13] focus on direct miRNA electrochemical biosensors based on redox markers with different designs, such as DNA-intercalating redox systems, redox catalysts as well as free redox indicators. The authors discuss the advantages and drawbacks of these approaches, presenting the state of the art and the challenges still open to improve validation, clinical application and possible commercialization of electrochemical biosensors for the detection of circulating miRNA.

Increasing attention is paid to the development of the biorecognition element able to promote a wider applicability of electrochemical biosensors. In their review, Palchetti and coworkers [14] present the state of the art and prospects on a key point crucial for the future of bioanalytical sensing, which is the development of electrochemical biosensors based on synthetic peptides. The advantages of easily synthetized peptides vs. antibodies are evident in terms of cost effectiveness and high yields. The authors discuss the different roles of peptides in the design of electrochemical biosensors, from their use as antifouling agents to their role in the development of catalytic and affinity electrochemical biosensors. Moreover, the authors discuss and compare the procedures used for the selection and synthesis of peptides as well as the different electrochemical detection strategies based on both label and label-free approaches, together with application for clinical diagnostics.

The drastic effect of viral infections and their transmission at an alarming rate has highlighted the need for advanced and rapid diagnostic techniques, as dramatically demonstrated by the COVID-19 pandemic. In their review, Arduini and coworkers [15] analyze the most recent achievements in the field of the quick detection of SARS-CoV-2 with biosensors. At first, the article presents and discusses the guidelines on COVID-19 for in vitro diagnostic tests and their performance, developed by the European Commission in 2020. Afterwards, the review examines technical and scientific aspects of the different biosensors developed up to the publication of the review, focusing on biosensors designed for the quantitative analysis of SARS-CoV-2 antigens, as tested on real matrices, such as nasopharynx swabs, saliva, serum and droplets. A critical comparison between biosensors able to provide quantitative responses vs. qualitative tests is presented. Finally, the authors discuss the pros and cons of available analytical tools to address a feasible strategy for fabricating an ideal biosensor suitable for the specific detection of SARS-CoV-2, hopefully offering the possibility of a wide range use and commercialization.

Thanking all the authors for their contributions, we hope that the articles presented in this Special Issue can offer a stimulating panorama of some of the open challenges for advancing research in the field of electrochemical and opto-electrochemical biosensors, in particular for the detection of viruses and disease markers. In our opinion, these contributions can constitute a useful starting point for further development in this important field, with a focus on some specific areas. Moreover, they show that when biochemistry, electrochemistry and analytical chemistry meet, it is possible to offer at least a partial answer to the need for real improvements in the quality of life and public health.

## Data Availability

Detailed information is available on request to the corresponding author.
